# Supplementary material for: Neuronal fragile X mental retardation protein activates glial insulin receptor mediated PDF-Tri neuron developmental clearance
Source: Nat Commun. 2021 Feb 19;12:1160. doi: 10.1038/s41467-021-21429-4 (PMC7896095; doi:10.1038/s41467-021-21429-4)
Supplement: Supplementary file 1 — Supplementary Information [file 41467_2021_21429_MOESM1_ESM.pdf]

Neuronal Fragile X Mental Retardation Protein activates glial insulin receptor mediated PDF-Tri neuron developmental clearance

Dominic J. Vita[1], Cole J. Meier[1] and Kendal Broadie[1,2,3*]

[1]Department of Biological Sciences, Vanderbilt University, Nashville, Tennessee, 37235, USA
[2]Kennedy Center for Research on Human Development, Nashville, Tennessee 37235, USA
[3]Vanderbilt Brain Institute, Vanderbilt University Medical Center, Nashville, Tennessee, 37235, USA

Supplemental Data

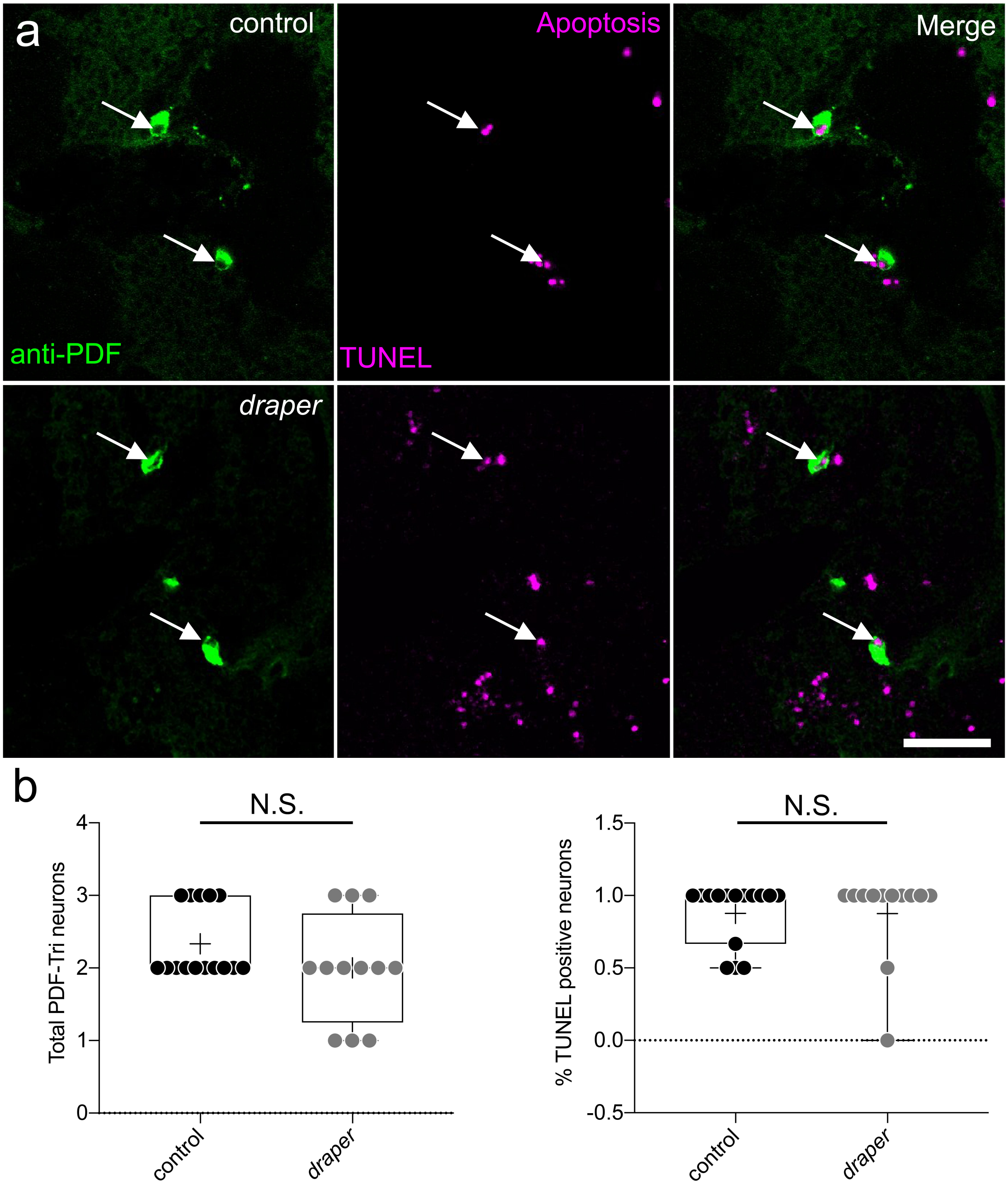

Supplemental Figure 1: *draper* null mutants show TUNEL-positive PDF-Tri neuron apoptosis

a. Representative central brain PDF-Tri region images (0 DPE) labeled with anti-PDF (green, left), TUNEL (magenta, middle) and the merge (right) in control (w^{1118}, top) and *draper* null mutant ($draper^{\Delta 5}$, bottom). Arrows indicate TUNEL positive PDF-Tri neuron cell bodies. Scale bar: 25μm. b. Quantification of the total number of PDF-Tri neuron cell bodies (left) and the percentage of TUNEL positive PDF-Tri neurons (right). Box and whisker plots show quartiles with max and min values, with plus sign indicating the mean. Total PDF-Tri neurons: Two-sided Mann-Whitney, p=0.763, 0.878±0.0551 n=15 control, 0.875±0.0897 n=12 *draper*. TUNEL positive neurons: Two-sided Mann-Whitney, p=0.227, 2.333±0.1260 n=15 control, 2.000±0.213 n=12 *draper*. Sample size is n=number of animals. Significance: p>0.05 (not significant, N.S.). Source data for this figure are provided in Source Data file.

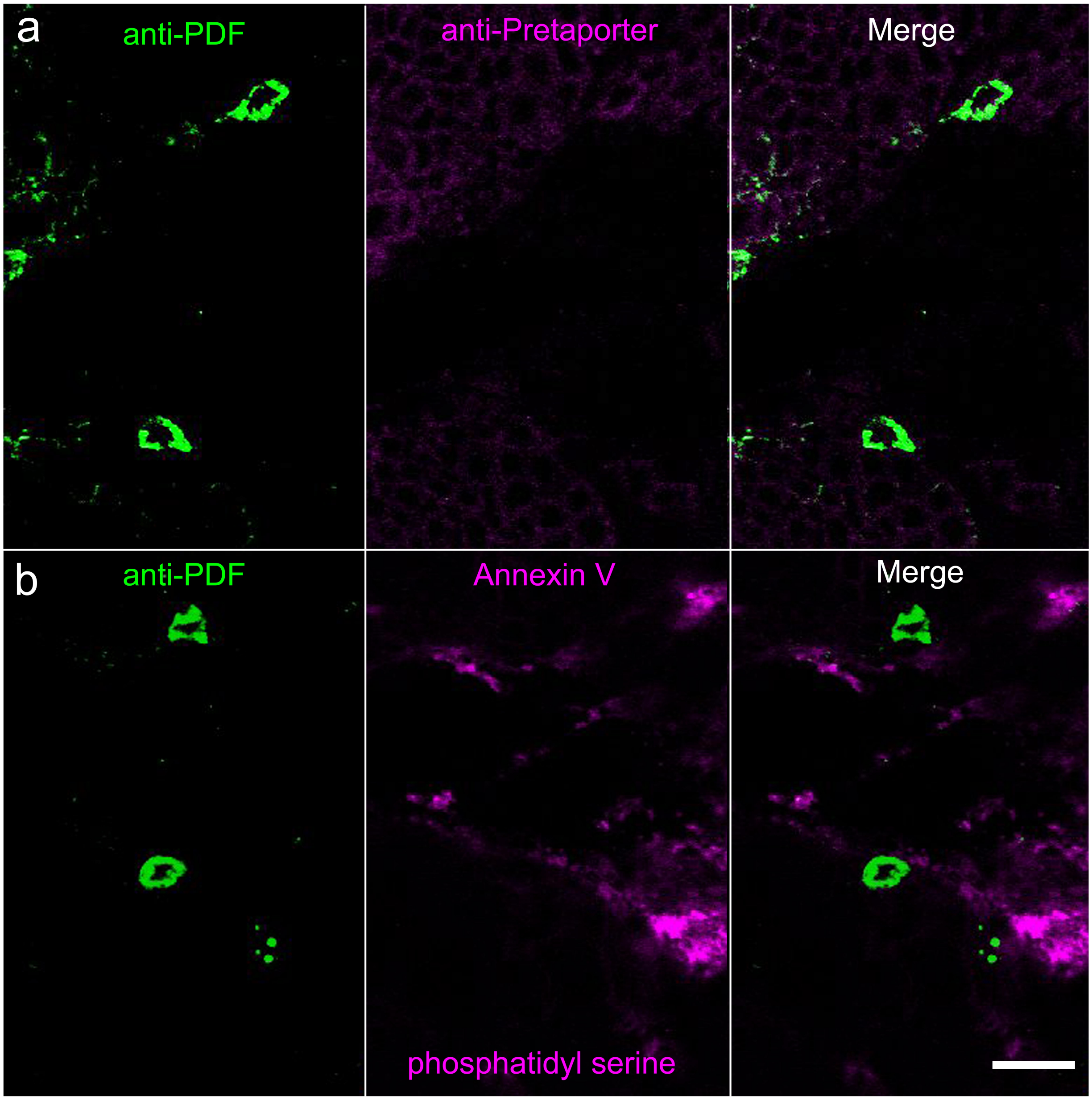

Supplemental Figure 2: PDF-Tri neurons lack candidate cell death/phagocytosis markers

a. Representative central brain PDF-Tri region images (0 DPE) labeled with anti-PDF (green, left), anti-Pretaporter (magenta, middle), and the merge (right). No enrichment is observed in PDF-Tri neurons. b. The same brain regions labeled for anti-PDF (green, left), phosphatidylserine (Annexin V, magenta, middle), and the merge (right). No enrichment is observed within PDF-Tri neurons. Scale bar: 10μm. Images are representative of two independent experiments.

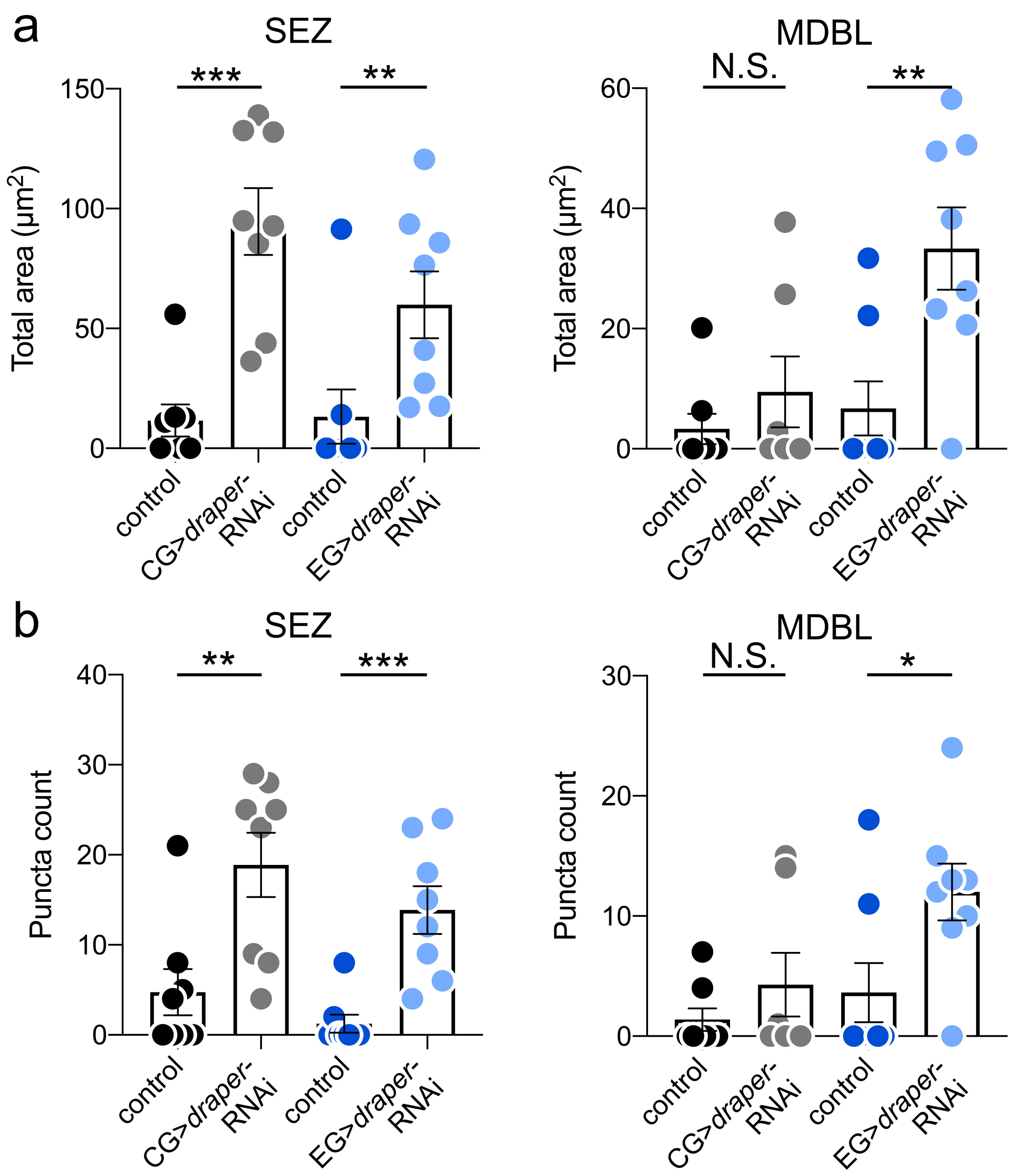

Supplemental Figure 3: Glial class PDF-Tri neuron clearance in spatially restricted domains
a. Quantification of anti-PDF area (5 DPE) in the proximal subesophageal zone (SEZ, left) and the distal medial bundle (MDBL, right) from the brains imaged in main text Figure 4a. SEZ: CG, two-sided Mann-Whitney, p=0.0003, 5.236±2.481 n=7 control, 94.62±13.93 n=8 *draper*-RNAi; EG, two-sided Mann-Whitney, p=0.0034, 13.19±11.31 n=8 control, 58.98±13.89 n=8 *draper*-RNAi. MDBL: CG, two-sided Mann-Whitney, p=0.4126, 3.305±2.524 n=8 control, 9.466±5.906 n=7 *draper*-RNAi; EG, two-sided t-test, p=0.0059, 6.738±4.501 n=8 control, 33.33±6.844 n=8 *draper*-RNAi. b. Quantification of PDF+ puncta (5 DPE) in the SEZ (left) and MDBL (right) from the same series of experiments. SEZ: CG, two-sided Mann-Whitney, p=0.0020, 2.429±1.232 n=7 control, 18.88±3.573 n=8 *draper*-RNAi; EG, two-sided Mann-Whitney, p=0.0006, 1.25±0.9955 n=8 control, 13.88±2.642 n=8 *draper*-RNAi. MDBL: CG, two-sided Mann-Whitney, p=0.4126, 1.375±0.9437 n=8 control, 4.286±2.643 n=7 *draper*-RNAi; EG, two-sided Mann-Whitney, p=0.00373, 3.625±2.464 n=8 control, 12.00±2.360 n=8 *draper*-RNAi. The cortex glia (CG) transgenic driver control (*R54H02*-Gal4/+) and CG>*draper*-RNAi experimental (*R54H02*-Gal4>*draper*-RNAi) are shown in black/grey, respectively. The ensheathing glia (EG) driver control (*R56F03*-Gal4/+) and EG>*draper*-RNAi experimental (*R56F03*-Gal4>*draper*-RNAi) are shown in dark/light blue, respectively. Scatter plots show mean ± SEM. Sample size is n=number of animals. Significance: p>0.05 (not significant N.S.), p<0.05 (*), p<0.01 (**) and p<0.001 (***). Source data for this figure are provided in Source Data file.

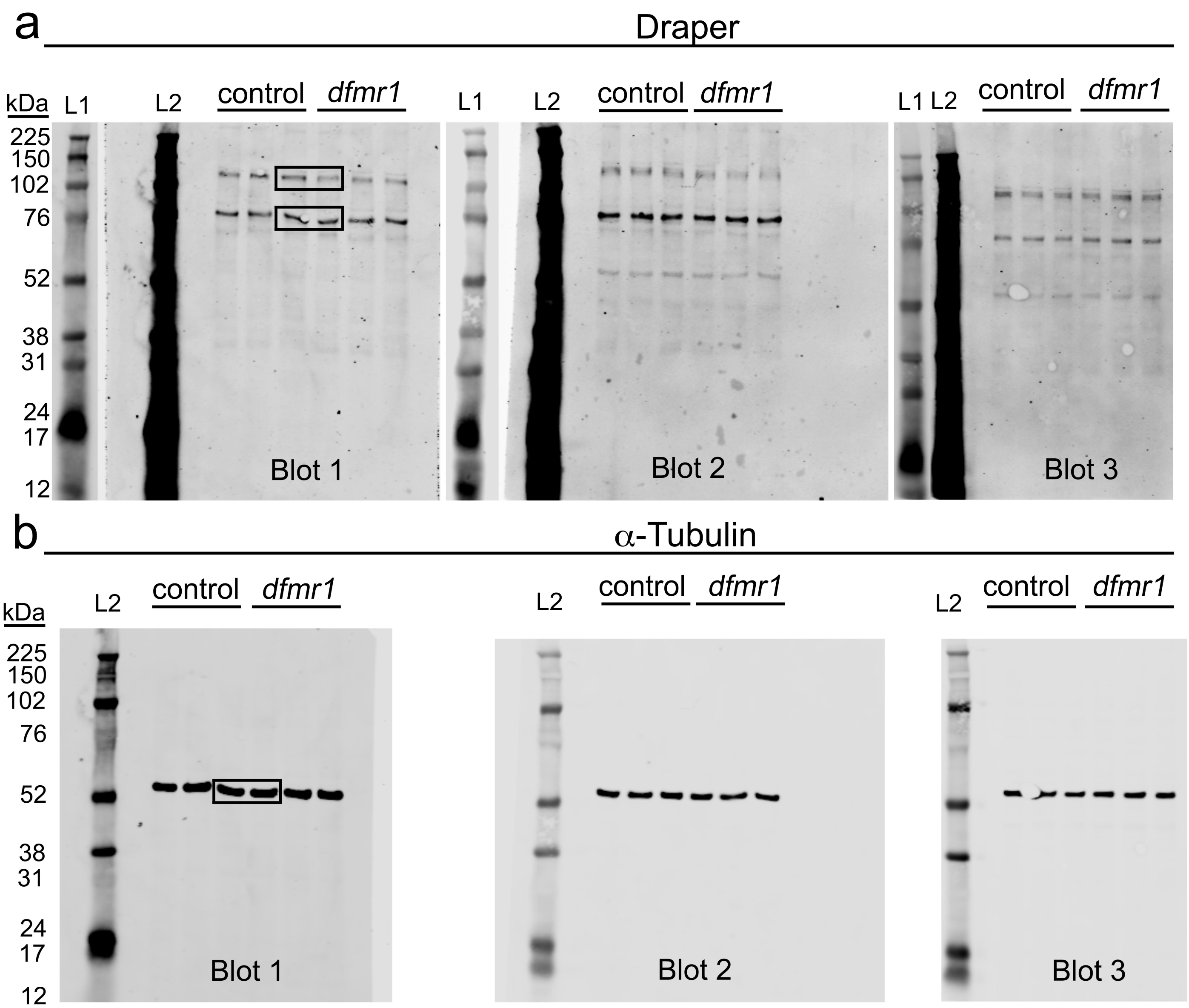

Supplemental Figure 4: Full Western blots from Figure 6a in the main text
Western blots of whole brain lysates (2 brains per lane, 0 DPE) for control (w^{1118}) and *dfmr1* null mutants (w^{1118}; $dfmr1^{50M}$). a. Anti-Draper Western blots with molecular weights listed to the left (kDa). L1 indicates ladder with reduced brightness to show bands. L2 indicates ladder with identical brightness/contrast settings as anti-Draper probe. b. Anti-α-tubulin with molecular weights listed to the left (kDa). L2 indicates ladder with identical brightness/contrast settings as the probe. The Western blot number is indicated, matching Draper and α-tubulin blots. Boxes indicate bands in main text.

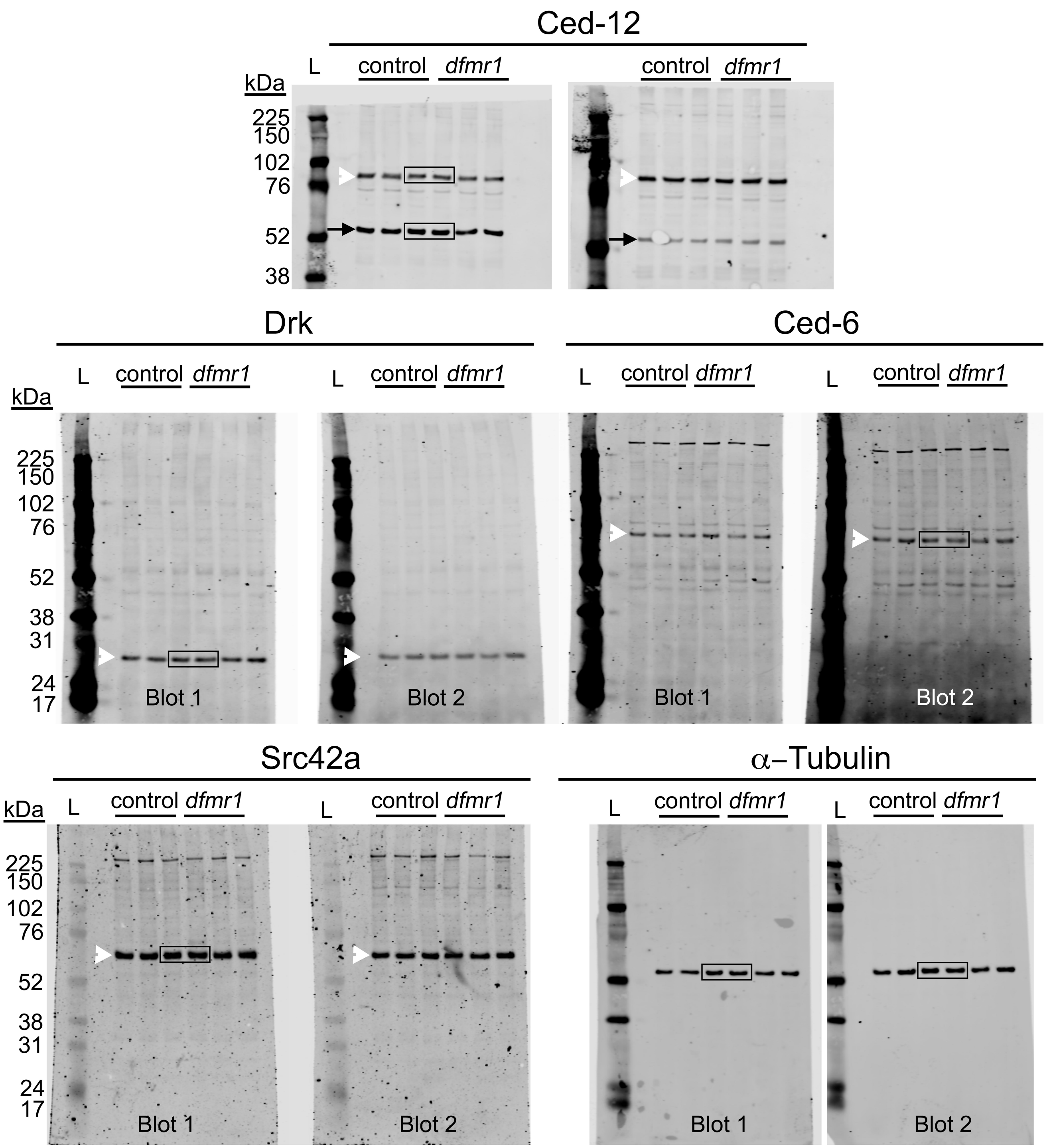

Supplemental Figure 5: Full Western blots from Figure 6b in the main text
Western blots of whole brain lysates (2 brains per lane, 0 DPE) for control (*w^{1118}*) and *dfmr1* null mutants (*w^{1118}*; *$dfmr1^{50M}$*). Molecular weights to the left (kDa), ladder (L) indicated and probes designated above. Top: Anti-Ced-12 Western blots (white arrowhead) with α-tubulin (black arrow). Middle: Anti-Drk (left, white arrowhead) and anti-Ced-6 (right, white arrowhead) Western blots. Bottom: Anti-Src42a (left, white arrowhead) and Anti-α-tubulin (right, white arrowhead) Western blots. The Western blot number is indicated in each case. Boxes indicate bands in main text.

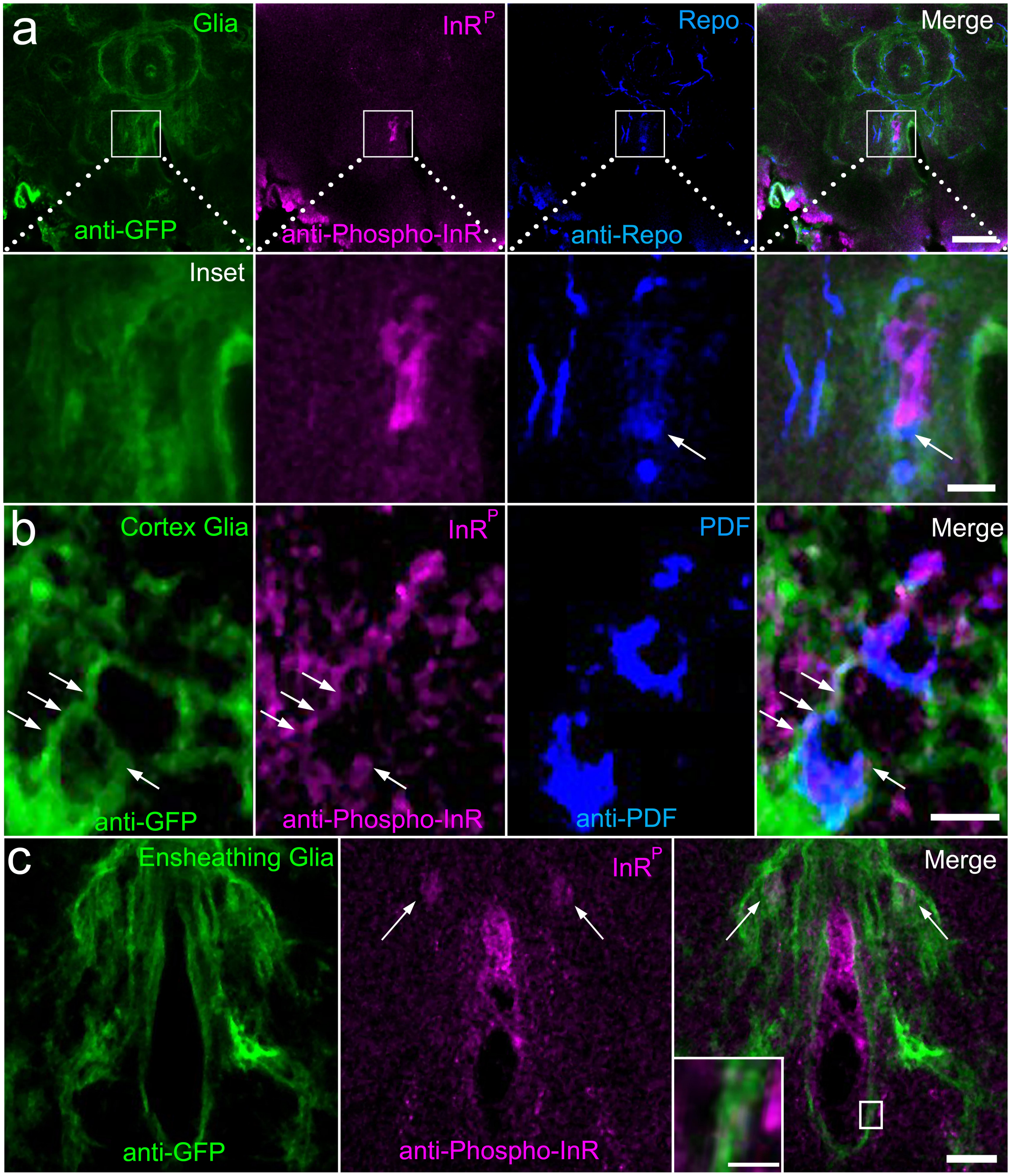

Supplemental Figure 6: InR^P labeling with anti-Repo glial and glial class GFP markers

a. Central brain slices (2 DPE) with *repo*-Gal4 driven mCD8::GFP in glia (green) co-labeled with anti-InR^P (magenta) and anti-Repo (blue). Top: Lower magnification images with the merge (right). Scale bar: 40μm. Bottom: High magnification of boxed region in top panel, showing co-localization. Arrow indicates Repo+ cell. Scale bar: 10μm. b. Central brain slices (0 DPE) with mCD8::GFP (green) driven in cortex glia via *R54H02*-Gal4, co-labeled with anti-InR^P (magenta) and anti-PDF (blue), and the merge (right). Arrows indicate InR^P+ glial membranes adjacent to PDF-Tri cell body. Scale bar: 10μm. c. Central brain slices (2 DPE) with mCD8::GFP (green) driven in ensheathing glia via *R56F03*-Gal4, co-labeled with anti-InR^P (magenta), and the merge (right). White arrows indicate InR^P+ cell bodies. Scale bar: 25μm. Inset is enlarged boxed region. Scale bar: 6μm. Images are representative of two independent experiments.

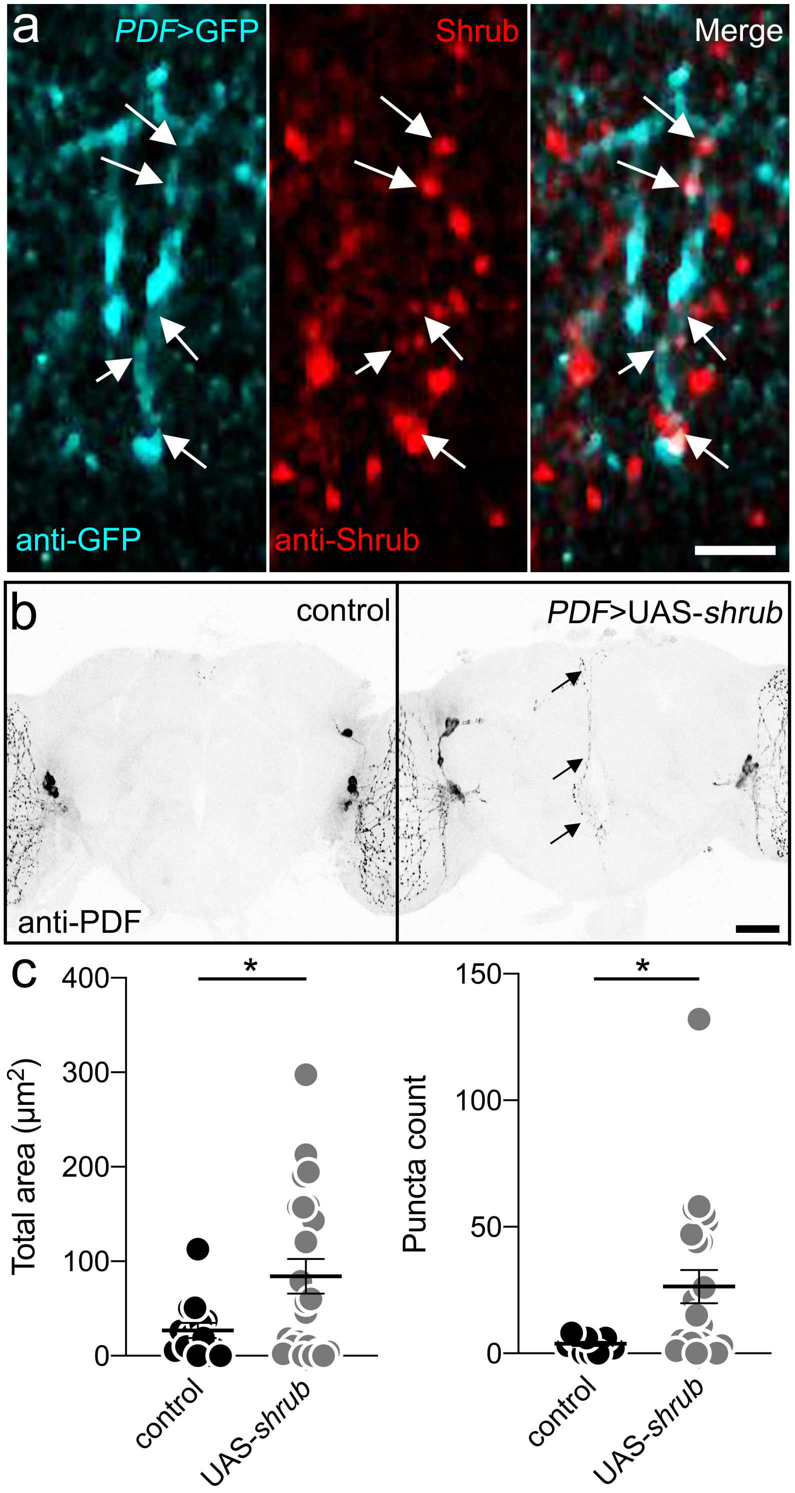

Supplemental Figure 7: Shrub gain-of-function (GOF) roles in PDF-Tri neuron clearance

a. Representative central brain PDF-Tri region images (2 DPE) with *PDF*-Gal4>mCD8::GFP (anti-GFP, cyan, left) and anti-Shrub (red, middle) in PDF-Tri neuron processes, with the merge (right). Arrows indicate thinned PDF-Tri neuron domains co-localized with anti-Shrub puncta. Scale bar: 5μm. Images are representative of two independent experiments. b. Whole brains (5 DPE) labeled with anti-PDF in transgenic control (*PDF*-Gal4/+, left) and PDF-Tri targeted Shrub overexpression (*PDF*-Gal4>UAS-*shrub*, right). PDF-Tri neurons (arrows) absent in controls, persist when Shrub is overexpressed. Scale bar: 50μm. c. Quantification of PDF area (left) and PDF+ puncta (right). Area: Two-tailed t-test, p=0.0.0248, 26.85±8.134 n=14 control, 84.22±18.34 n=23 UAS-*shrub*. Puncta: Mann-Whitney, p=0.0384, 3.769±0.7775 n=13 control, 26.39±6.579 n=23 UAS-*shrub*. Scatter plots show mean ± SEM. Sample size is n=number of animals. Significance shown: p<0.05 (*). Source data for this figure are provided in Source Data file.

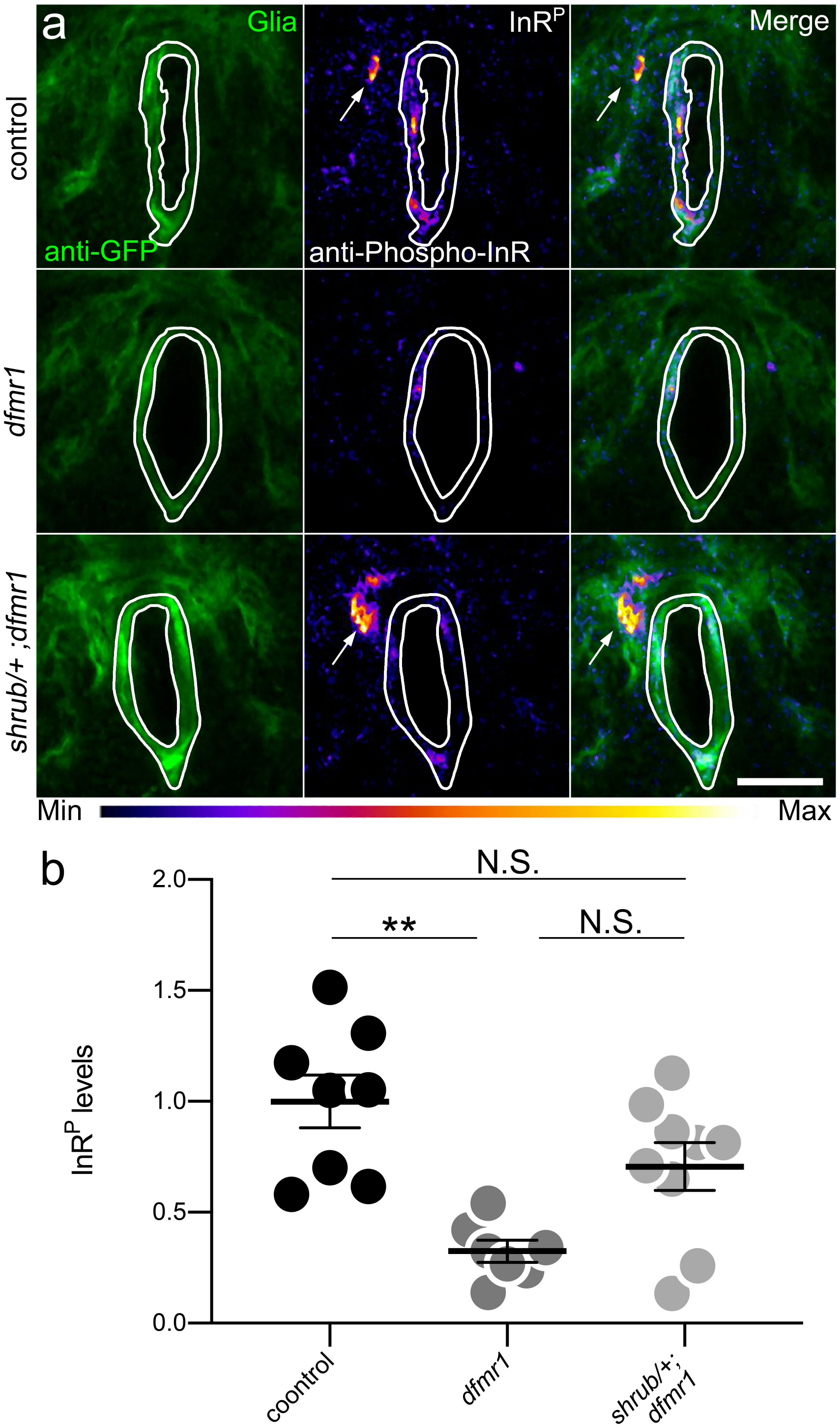

Supplemental Figure 8: FMRP and Shrub interact to drive glial insulin receptor activation

a. Whole brains (2 DPE) co-labeled for glial targeted mCD8::GFP (anti-GFP, green, left), anti-phosphorylated insulin receptor (InR^P, heat-map intensity, middle) and the merged image (right) in transgenic control (UAS-mCD8::GFP/+; *repo*-Gal4/+, top), *dfmr1* null mutant (UAS-mCD8::GFP/+; *dfmr1*^{50M}, *repo*-Gal4/d*fmr1*^{50M}, middle) and heterozygote null *shrub*/+ in the *dfmr1* null (UAS-mCD8::GFP/*shrub*^{4}; *dfmr1*^{50M}, *repo*-Gal4/d*fmr1*^{50M}, bottom). The white outline indicates glial area used for InR^P intensity measurements, and arrows indicate glial cell bodies. Scale bar: 25μm. The range indicator below shows InR^P intensity levels. b. Quantification of normalized InR^P levels from the images in panel a. Kruskal-Wallis followed by Dunn's multiple comparison test, p=0.0030, 1.00±0.1200 n=8 control, 0.3246±0.04922 n=7 *dfmr1*; p=0.5309, 1.00±0.1200 n=8 control, 0.7064±0.1076 n=9 *shrub;* p=0.1138, 0.3246±0.04922 n=7 *dfmr1*, 0.7064±0.1076 n=9 *shrub.* Scatter plots show mean ± SEM. Significance shown: p>0.05 (not significant N.S.) and p>0.01 (**). Source data for this figure are provided in Source Data file.

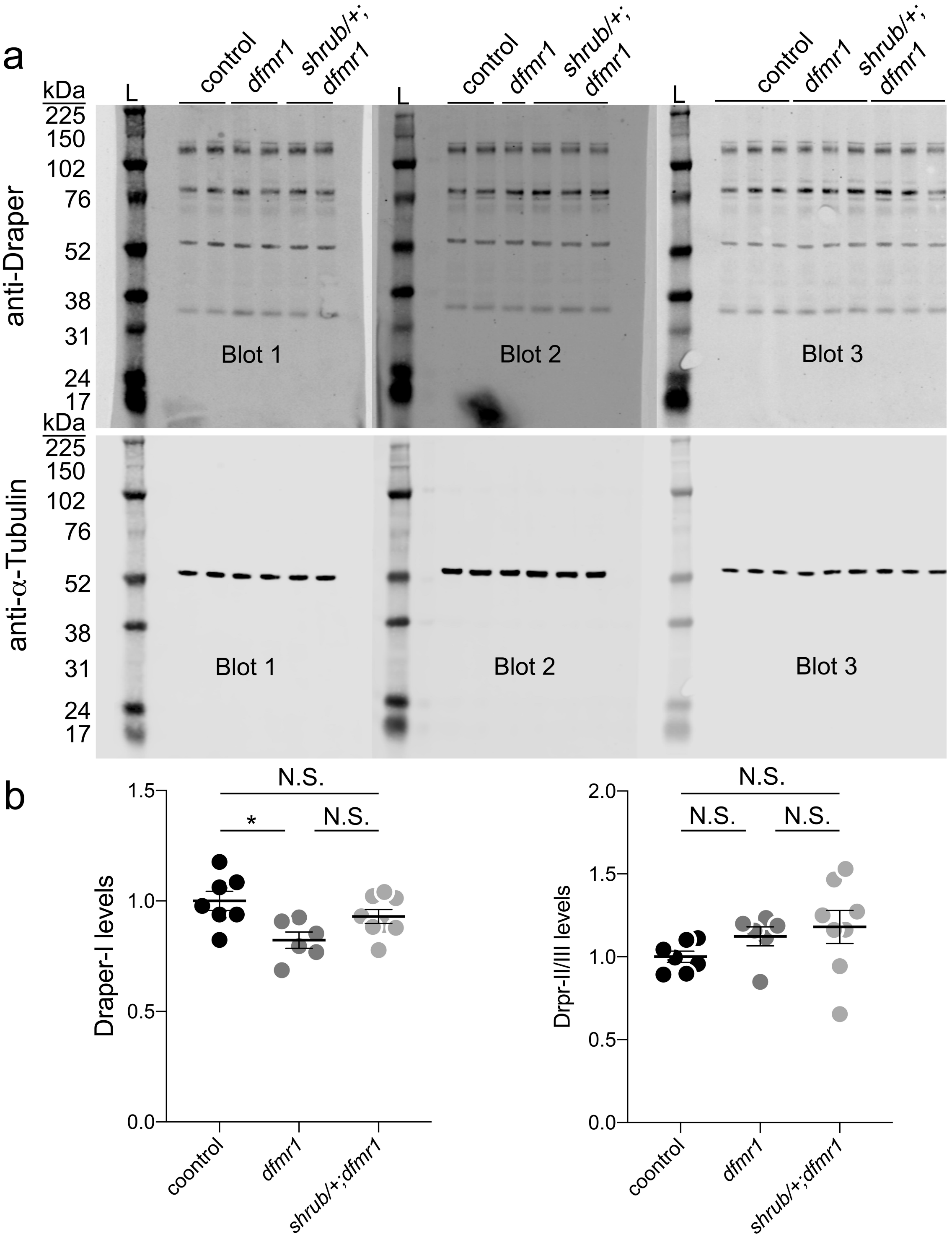

Supplemental Figure 9: FMRP and ESCRT-III Shrub interact to facilitate Draper expression

a. Full Western blots of whole brain lysates (0 DPE, 2 brains per lane), probing for anti-Draper (top) and anti-α-tubulin (bottom) in the genetic background control (*w^{1118}*), *dfmr1* null mutant (*w^{1118}*; *$dfmr1^{50M}$*), and heterozygous null *shrub*/+ in the *dfmr1* null mutant (*w^{1118}*; *$shrub^{4}$*/+; *$dfmr1^{50M}$*). The molecular weights are shown to the left (kDa), ladder (L) indicated, and genotypes shown at the top. b. Quantification of Draper-I (left) and Draper-II/III (right) bands normalized to α-tubulin Draper-I: Kruskal-Wallis followed by Dunn's multiple comparison test, p=0.0160, 1.00±0.1162 n=7 control, 0.8227±0.03697 n=6 *dfmr1*; p=0.6266, 1.00±0.1162 n=7 control, 0.9295±0.03188 n=8 *shrub;* p=0.2870, 0.8227±0.03697 n=6 *dfmr1*, 0.9295±0.03188 n=8 *shrub.* Sample size is n=number of independent protein extractions (2 brains per extraction). Scatter plots show mean ± SEM. Significance shown: p>0.05 (not significant, N.S.) and p<0.05 (*). Source data for this figure are provided in Source Data file.
