# Supplementary material for: Neuronal fragile X mental retardation protein activates glial insulin receptor mediated PDF-Tri neuron developmental clearance
Source: Nat Commun. 2021 Feb 19;12:1160. doi: 10.1038/s41467-021-21429-4 (PMC7896095; doi:10.1038/s41467-021-21429-4)
Supplement: Supplementary file 3 — Reporting Summary [file 41467_2021_21429_MOESM3_ESM.pdf]

nature research

Corresponding author(s): Kendal Broadie

Last updated by author(s): 2021/1/3

Reporting Summary

Nature Research wishes to improve the reproducibility of the work that we publish. This form provides structure for consistency and transparency in reporting. For further information on Nature Research policies, see our Editorial Policies and the Editorial Policy Checklist.

Statistics

For all statistical analyses, confirm that the following items are present in the figure legend, table legend, main text, or Methods section.

n/a	Confirmed	
☐	☒	The exact sample size (n) for each experimental group/condition, given as a discrete number and unit of measurement
☐	☒	A statement on whether measurements were taken from distinct samples or whether the same sample was measured repeatedly
☐	☒	The statistical test(s) used AND whether they are one- or two-sided<br>*Only common tests should be described solely by name; describe more complex techniques in the Methods section.*
☒	☐	A description of all covariates tested
☐	☒	A description of any assumptions or corrections, such as tests of normality and adjustment for multiple comparisons
☐	☒	A full description of the statistical parameters including central tendency (e.g. means) or other basic estimates (e.g. regression coefficient) AND variation (e.g. standard deviation) or associated estimates of uncertainty (e.g. confidence intervals)
☐	☒	For null hypothesis testing, the test statistic (e.g. F, t, r) with confidence intervals, effect sizes, degrees of freedom and P value noted<br>*Give P values as exact values whenever suitable.*
☒	☐	For Bayesian analysis, information on the choice of priors and Markov chain Monte Carlo settings
☒	☐	For hierarchical and complex designs, identification of the appropriate level for tests and full reporting of outcomes
☒	☐	Estimates of effect sizes (e.g. Cohen's d, Pearson's r), indicating how they were calculated

Our web collection on statistics for biologists contains articles on many of the points above.

Software and code

Policy information about availability of computer code

Data collection	All imaging was done with a Zeiss 510 META laser-scanning confocal with 20X air or 40X oil immersion objectives. All images were collected with 1072X1072 resolutions and optical thickness set via the optimization function in Zen Black Edition version 8.1 software. Image parameters were kept constant across conditions within replicates. Western blot images were obtained via Li-Cor Odyssey imager.
Data analysis	All measurements for fluorescent images were performed in FIJI including areas, puncta count, and intensity. Western blots were measured with Image Studio Lite. All data analysis was performed in GraphPad Prism 8.

For manuscripts utilizing custom algorithms or software that are central to the research but not yet described in published literature, software must be made available to editors and reviewers. We strongly encourage code deposition in a community repository (e.g. GitHub). See the Nature Research guidelines for submitting code & software for further information.

Data

Policy information about availability of data

All manuscripts must include a data availability statement. This statement should provide the following information, where applicable:

- Accession codes, unique identifiers, or web links for publicly available datasets
- A list of figures that have associated raw data
- A description of any restrictions on data availability

Datasets generated for this study are available upon request from the corresponding author. Source data is available as source data file. Unaltered blots are supplemental figures.

Field-specific reporting

Please select the one below that is the best fit for your research. If you are not sure, read the appropriate sections before making your selection.

- [x] Life sciences
- [ ] Behavioural & social sciences
- [ ] Ecological, evolutionary & environmental sciences

For a reference copy of the document with all sections, see nature.com/documents/nr-reporting-summary-flat.pdf

Life sciences study design

All studies must disclose on these points even when the disclosure is negative.

Sample size	No statistical methods were used to determine sample size. Typical sample size in the field is 5-20. With this in mind, sample size was determined based on our initial observations and previous reports Gatto and Broadie (2011). With the exception of Western blots, sample size N refers to number of brains. For Western blots N refers to number of independent protein extractions which each consisted of 2 brains.
Data exclusions	All datasets in all experiments were subject to a ROUT outlier test to remove any outliers that could impact data interpretation. The ROUT outlier test is an established means to remove outlier data as recommended by GraphPad Prism software. The decision to perform this test on every dataset in this study was made a priori to ensure analysis was done in an unbiased fashion.
Replication	All experiments were replicated multiple times, spaced over time, with independent samples for each replicate. All attempts at replication were successful.
Randomization	Flies were sampled randomly for each experiment.
Blinding	All data sets were analyzed blind via renaming image files. Western blots were run with all conditions simultaneously with each blot/image containing all conditions in parallel at the same time.

Reporting for specific materials, systems and methods

We require information from authors about some types of materials, experimental systems and methods used in many studies. Here, indicate whether each material, system or method listed is relevant to your study. If you are not sure if a list item applies to your research, read the appropriate section before selecting a response.

Materials & experimental systems

n/a	Involved in the study
[ ]	[x] Antibodies
[x]	[ ] Eukaryotic cell lines
[x]	[ ] Palaeontology and archaeology
[ ]	[x] Animals and other organisms
[x]	[ ] Human research participants
[x]	[ ] Clinical data
[x]	[ ] Dual use research of concern

Methods

n/a	Involved in the study
[x]	[ ] ChIP-seq
[x]	[ ] Flow cytometry
[x]	[ ] MRI-based neuroimaging

Antibodies

Antibodies used

Primary Antibodies with commercial availability:
1. Rabbit anti GFP (Abcam 290)
2. Mouse anti PDF (DSHB PDF C7)
3. Mouse anti Draper (DSHB 8A1)
4. Rabbit anti phospho InR beta (Cell Signaling Tyr1146)
5. Chicken anti GFP (Abcam, AB13970)
6. Mouse anti Repo (DSHB 8D12)

Primary Antibodies without commercial availability:
1. Rabbit anti Shrub (gift from Fen-Bioa Gao's lab, Reference-Sweeney et al 2006)
2. Goat anti Ced-12 (gift from Erika Geisbrecht, Reference-Geisbrecht et al 2008)
3. Rat anti Ced-6 (gift from takeshi Awasaki, Reference-Awasaki et al 2006)
4. Chicken anti Src42a (gift from Shigeo Hayashi, Reference-Shindo et al 2008)
5. Rabbit anti Drk (gift from Efthimios Skoulakis, Reference-Moressis et al 2009)
6. Rat anti Pretaporter (gift from Yoshinobu Nakanishi, Reference-Kuraishi et al 2009)

Secondary Antibodies:
1. AlexaFluor 488 goat anti rabbit (Invitrogen A10667)

2. AlexaFluor 568 goat anti mouse (Invitrogen A11004)
3. AlexaFluor 633 goat anti mouse (Invitrogen A21050)
4. AlexaFluor 568 goat anti rabbit (Life technologies A11011)
5. AlexaFluor 800 goat anti mouse (Invitrogen A32703)
6. AlexaFluor 680 donkey anti goat (Invitrogen A21084)
7. AlexaFluor 680 goat anti rat (Invitrogen A21069)
8. AlexaFluor 790 goat anti chicken (Abcam 175787)
9. AlexaFlour 488 goat anti chicken (Invitrogen A11039)
10. AlexaFluor 680 goat anti rabbit (Invitrogen A27042)
12. AlexaFluor 488 goat anti mouse (Invitrogen A10667)

Validation

Primary Antibodies:
1. Rabbit anti GFP (Abcam 290)
https://www.abcam.com/gfp-antibody-chip-grade-ab290.html
Information as listed on the website written above:
Product name
Anti-GFP antibody
Description
Rabbit polyclonal to GFP
Host species
Rabbit
Specificity
Anti-GFP antibody (ab290) is a highly versatile antibody that gives a stronger signal than other anti-GFP antibodies available. On Western blot the antibody detects the GFP fraction from cell extracts expressing recombinant GFP fusion proteins and has also been shown to be useful on mouse sections fixed with formalin. In Immunocytochemistry, the antibody gives a very good signal on recombinant YES-GFP chimeras expressed in COS cells (McCabe et al. 1999 and figure below). It is routinely used in Immunoprecipitation (IP) and IP-Western protocols and has been used successfully in HRP Immunohistochemistry at 1:200 on whole-mount mouse embryos.
This antibody is reactive against all variants of Aequorea victoria GFP such as S65T-GFP, RS-GFP and EGFP.
Tested applications
Suitable for: ELISA, IHC-FrFl, Electron Microscopy, IHC-FoFr, ICC, IHC-P, IHC-Fr, IP, WB
Species reactivity
Reacts with: Species independent
Immunogen
Recombinant full length protein corresponding to GFP.
Database link: P42212
Validated in: Flow Cyt, ELISA, ICC/IF, ChIP, IHC-FrFl, ChIP/Chip, IHC - Wholemount, Electron Microscopy, IHC-FoFr, ICC, IHC-P, IHC-Fr, IP, WB.
Reference: Golovin et al. J Neurosci. 39(16):2995-3012. (2019).
Data in this study Fig 1, 3a, 9a. Supp Fig 6, 7, 8.

2. Mouse anti PDF (DSHB PDF C7)
https://dshb.biology.uiowa.edu/PDF-C7
Information as listed on the website written above:
Antigen: Pigment-dispersing factor neuropeptide
Hybridoma Cells Available: Yes
Antigen Species: Drosophila
Isotype: MIgG2b, kappa light chain
Host Species: mouse Depositors
Positive Tested Species Reactivity: Cabbage root fly, Cockroach, Drosophila, Mosquito
Depositors Notes: Fusion date: 2005. Recognizes Drosophila larval and adult brains.
Antigen Molecular Weight: Predicted: PDF, 2kDa; PDF precursor, 11.5kDa
Gene: Pdf
Immunogen: amidated pigment dispersing factor neuropeptide
Alternate Gene Names: Dmel\CG6496; Drm-pdf; Drm-PDF; PAP; cPDH; PDH
Clonality: Monoclonal
Epitope Mapped: Yes
Myeloma Strain: SP2-0/Ag14
Epitope Location or Sequence: NSELINSLLSLPKNMNDA-NH2
Uniprot ID: O96690
Immunogen Sequence: NSELINSLLSLPKNMNDA-NH2
Entrez Gene ID: 43193
Antibody Registry ID: AB_760350 AB_2315084
Additional Information: The neuropeptide PDF represents the C-terminus portion (aa 83-100) of the Protein PDF [Uniprot O96690]. The neuropeptide PDF regulates circadian loco motor rhythms.
Recommended Applications: Immunofluorescence, Immunohistochemistry
Recommended applications immunofluorescence and immunohistochemistry.
References: Cyran et al. J Neurosci. Jun 1;25(22):5430-7(2005). Selcho et al. J Comp Neurol. 526(8):1307-1328 (2018).

Data in this study Fig 1, 2, 3b-d, 4, 5, 6c, 7, 8, 9b, 10b. Supp Fig 1, 2, 6b, 7b

3. Mouse anti Draper (DSHB 8A1)
https://dshb.biology.uiowa.edu/Draper-8A1
Antigen: Draper
Hybridoma Cells Available: Yes
Antigen Species: Drosophila melanogaster
Isotype: MIgG2a
Host Species: mouse
Positive Tested Species Reactivity: Drosophila Depositors
Notes: Fixation: 4% PFA for whole mount.
Antigen Molecular Weight: Predicted: Multiple isforms between 59 and 114 kDa
Gene: drpr
Immunogen: NPIVYNESLK
Alternate Gene Names: CG2086
Clonality: Monoclonal
Epitope Mapped: Yes
Myeloma Strain: SP2/0
Epitope Location or Sequence: NPIVYNESLK
Uniprot ID: Q9W0A0 Q9W0A1 M9PGU6 M9PDW5 M9PBI3 M9NEX8
Immunogen Sequence: Partial protein
Entrez Gene ID: 38218
Antibody Registry ID: AB_2618106
Additional Information: Draper is an engulfment receptor involved in cell death and phagocytosis. This antibody will recognize all Draper isoforms.
Recommended applications immunofluorescence and Western blot.
Reference: Musashe et al. Cell Rep 16(7):1838-50 (2016). Used this for immuno labeling of drosophila brains.
Data in this study Fig 6a. Supp Fig 4a, 6a, 9a.

4. Rabbit anti phospho InR beta (Cell Signaling Tyr1146)
https://www.cellsignal.com/products/primary-antibodies/phospho-igf-i-receptor-b-tyr1131-insulin-receptor-b-tyr1146-antibody/3021
Antibody was Validated by manufacturer with Western blot of extracts from 3T3-L1 adipocytes, untreated or insulin-treated, and IP.
Reference: Musashe et al. Cell Rep 16(7):1838-50 (2016). Further Validated in this reference via immunostaining in animals expressing insulin receptor knock down and show reduction in signal.
Data in this study Fig 9a. Supp Fig 6, 8.

5. Rabbit anti Shrub (gift from Fen-Bioa Gao's lab, Sweeney et al 2006)
Reference of generation: Sweeney et al. Curr Biol. 16(10):1006-11. Validated via western blot of Shrub null.
additional reference: Vita and Broadie Sci Rep. 7, Article number: 8683 (2017). Reducing Shrub levels resulted in reduced Shrub bands on Western blot.
Data in this study Fig 10a. Supp Fig 7a

6. Goat anti Ced-12 (gift from Erika Geisbrecht, Geisbrecht et al 2008)
Reference of generation: Geisbrecht et al. Dev Biol. 314(1):137-49 (2008). Validated via Western blot of HA-tagged Ced-12 after IP. Bands were further confirmed via mas spectrometry.
Data in this study Fig 6b.

7. Rat anti Ced-6 (gift from Takeshi Awasaki, Awasaki et al 2006)
Reference of generation: Awasaki et al. Neuron. 50(6):855-67. (2006). Validated via Western blot.
Data in this study Fig 6b.

8. Chicken anti Src42a (gift from Shigeo Hayashi, Shindo et al 2008)
Reference of generation: Shindo et al. Development. 135(7):1355-64. (2008). Validated via immunostaining in Src42a mutants. Also used in Western blots after IP.
Data in this study Fig 6b.

9. Rabbit anti Drk (gift from Efthimios Skoulakis, Moressis et al 2009)
Reference of generation: Moressis et al. J Neurosci. 29(8):2611-25 (2009). Validated via immunostaining of homozygous null embryos. Antibody also used in western blots.
Data in this study Fig 6b.

10. Chicken anti GFP (Abcam, AB13970)
https://www.abcam.com/gfp-antibody-ab13970.html
Product name
Anti-GFP antibody
Description
Chicken polyclonal to GFP
Host species

Chicken
Specificity
Our GFP antibody does cross-react with the many fluorescent proteins that are derived from the jellyfish Aequorea victoria. These are all proteins that differ from the original GFP by just a few point mutations (EGFP, YFP, mVenus, CFP, BFP etc.).
Tested applications
Suitable for: WB, ICC/IF
Immunogen
Recombinant full length protein corresponding to GFP.
Database link: P42212
Validated in: IHC-P, WB, ICC/IF, IHC-Fr, IHC-FoFr.
Reference: Yu X et al. Nat Commun 11:264 (2020).
Data in this study Supp Fig 7a.

11. Mouse anti Repo (DSHB 8D12)
https://dshb.biology.uiowa.edu/8D12-anti-Repo
Antigen: Repo; Reversed polarity protein
Hybridoma Cells Available: Yes
Antigen Species: Drosophila
Depositor: Goodman, C.
Isotype: MIgG1, kappa light chain
Host Species: mouse
Positive Tested Species Reactivity: Drosophila Depositors
Notes: Excellent marker for glial cells; stains the nuclei of all glia, except for the midline glia
Gene: Repo
Immunogen: Repo (a.a. 218-612)-6Xhistidine fusion protein.
Alternate Gene Names: AbRK2; Repo; REPO; rk2; RK2
Clonality: Monoclonal
Epitope Mapped: No
Myeloma Strain: P3 x 63 Ag8.653 Epitope Location or Sequence:
Uniprot ID: Q7KSE4 Immunogen Sequence:
Entrez Gene ID: 47285 Additional Characterization:
Antibody Registry ID: AB_528448 Additional Information:
Recommended Applications: Immunofluorescence, Immunohistochemistry
Reference: Alfonso, TB. and Jones, BW. Dev Biol. Aug 15;248(2):369-83 (2002).
Data in this study Supp Fig 6a.

12. Rat anti Pretaporter (gift from Yoshinobu Nakanishi, Kuraishi et al 2009)
Reference of generation: Kuraishi et al. EMBO J. 28, 3868–3878 (2009). Validated via Western blot with Pretaporter null.
Data in this study Supp Fig 2a.

Animals and other organisms

Policy information about studies involving animals; ARRIVE guidelines recommended for reporting animal research

Laboratory animals	Drosophila melanogaster. Males and females were used. Animals were aged to 0 days post eclosion (DPE), 2 DPE or 5 DPE as indicated in the text and figure legends.
Wild animals	No wild animals were used.
Field-collected samples	No field collection was performed.
Ethics oversight	No ethics oversight is required for Drosophila melanogaster studies.

Note that full information on the approval of the study protocol must also be provided in the manuscript.
